# Seagrass *Posidonia* is impaired by human-generated noise

**DOI:** 10.1038/s42003-021-02165-3

**Published:** 2021-06-15

**Authors:** Marta Solé, Marc Lenoir, Mercè Durfort, José-Manuel Fortuño, Mike van der Schaar, Steffen De Vreese, Michel André

**Affiliations:** 1 Laboratory of Applied Bioacoustics, Technical University of Catalonia, BarcelonaTech (UPC), Vilanova i la Geltrú, Barcelona Spain; 2 INSERM U.1051, Institute of Neurosciences of Montpellier, Montpellier, France; 3 Department of Cellular Biology, Faculty of Biology, University of Barcelona, Barcelona, Spain; 4 Electron Microscopy Laboratory, Institute of Marine Sciences, Spanish National Research Council (CSIC), Barcelona, Spain; 5 Department of Comparative Biomedicine and Food Science, University of Padua, Legnaro, Padua, Italy

**Keywords:** Ecosystem ecology, Plant physiology, Abiotic, Plant signalling, Plant symbiosis

## Abstract

The last hundred years have seen the introduction of many sources of artificial noise in the sea environment which have shown to negatively affect marine organisms. Little attention has been devoted to how much this noise could affect sessile organisms. Here, we report morphological and ultrastructural changes in seagrass, after exposure to sounds in a controlled environment. These results are new to aquatic plants pathology. Low-frequency sounds produced alterations in *Posidonia oceanica* root and rhizome statocysts, which sense gravity and process sound vibration. Nutritional processes of the plant were affected as well: we observed a decrease in the number of rhizome starch grains, which have a vital role in energy storage, as well as a degradation in the specific fungal symbionts of *P. oceanica* roots. This sensitivity to artificial sounds revealed how sound can potentially affect the health status of *P. oceanica*. Moreover, these findings address the question of how much the increase of ocean noise pollution may contribute in the future to the depletion of seagrass populations and to biodiversity loss.

## Abstract (web summary)

Solé et al. report morphological and ultrastructural changes in seagrass, after exposure to human generated noise. These data suggest that noise pollution can potentially affect the health status of seagrass and thereby contribute to the depletion of seagrass populations.

## Introduction

The extent to which noise in the sea affects marine ecosystems has become a topic of concern to the scientific community as well as to society at large^1,2^ A critical question is whether human-generated noise and other forms of energy may interfere with the normal behavior of marine organisms or cause them physical harm. Based on current knowledge, little doubt remains that acoustic noise from human activities can introduce unprecedented risks for key marine species, biodiversity, ecosystems, and overall ocean health^3–10^.

Indeed, considerable scientific attention has been devoted in the last decade to determining the sensitivity of aquatic animals to noise. Research has focused especially on cetaceans, pinnipeds^3–5^, and fish^6^ because these are known to possess hearing organs. Recent studies have also shown that artificial sounds affect cephalopods, cnidarians, and crustaceans even though they lack proper auditory receptors^8,11–13^. However, no study has addressed the sensitivity to noise and other forms of energy of sessile marine organisms, such as plants or coral reefs, whose immobility makes them highly susceptible to chronic effects. These marine organisms have sensory organs that are specialized in gravity perception, essential for detecting their natural substrate and with which inner structures could be affected by sound exposure.

Seagrass meadows considered an ocean equivalent to primary forests are the most widespread coastal ecosystems and rank among the most valuable biomes for the planet^14,15^. It provides essential ecosystem services such as carbon burial, nutrient cycling, erosion protection, biodiversity promotion, pathogen removal, and water quality improvement^16,17^. The family *Posidoniaceae* is the evolutionary oldest seagrass genus and its earliest fossil record dates back to the Cretaceous^15^.

Seagrasses belong to four families of the order *Alismatales*. These plants are the foundation of highly productive ecosystems along the coasts of all continents, except Antarctica^18^. Seagrasses are clonal plants fully adapted to a saline medium (marine ecosystem), where they complete their entire life cycle, including flowering, pollen transport, and seed germination. *P. oceanica* is a slow-growing seagrass, endemic to the Mediterranean Sea, which develops a deep network of roots and rhizomes that can extend several meters deep and be thousands of years and dominates shallow coastal landscapes^19^. *P. oceanica* forms extensive meadows that represent the climax communities in those areas^19^ and have shown to be highly vulnerable to small increases in mortality rate^20^. Excessive organic input, mechanical impacts, or regional climate warming could trigger such small increases, leading to a rapid loss in vegetation^20,21^. The worldwide rate of seagrass decline has been estimated to be 110 km^2^ per year since 1980^22^. In this context of vulnerability, additional external pressure could contribute to progressive seagrass depletion. Ship activity, coastal development building of groynes and seawalls, as well as associated dredging of channels and harbors has been correlated with most seagrass losses^20–22^. Interestingly, but not conclusive, a common factor among these human activities together with direct physical destruction of the plant environment is a massive introduction of anthropogenic sound sources into the ocean.

**Fig. 1:**
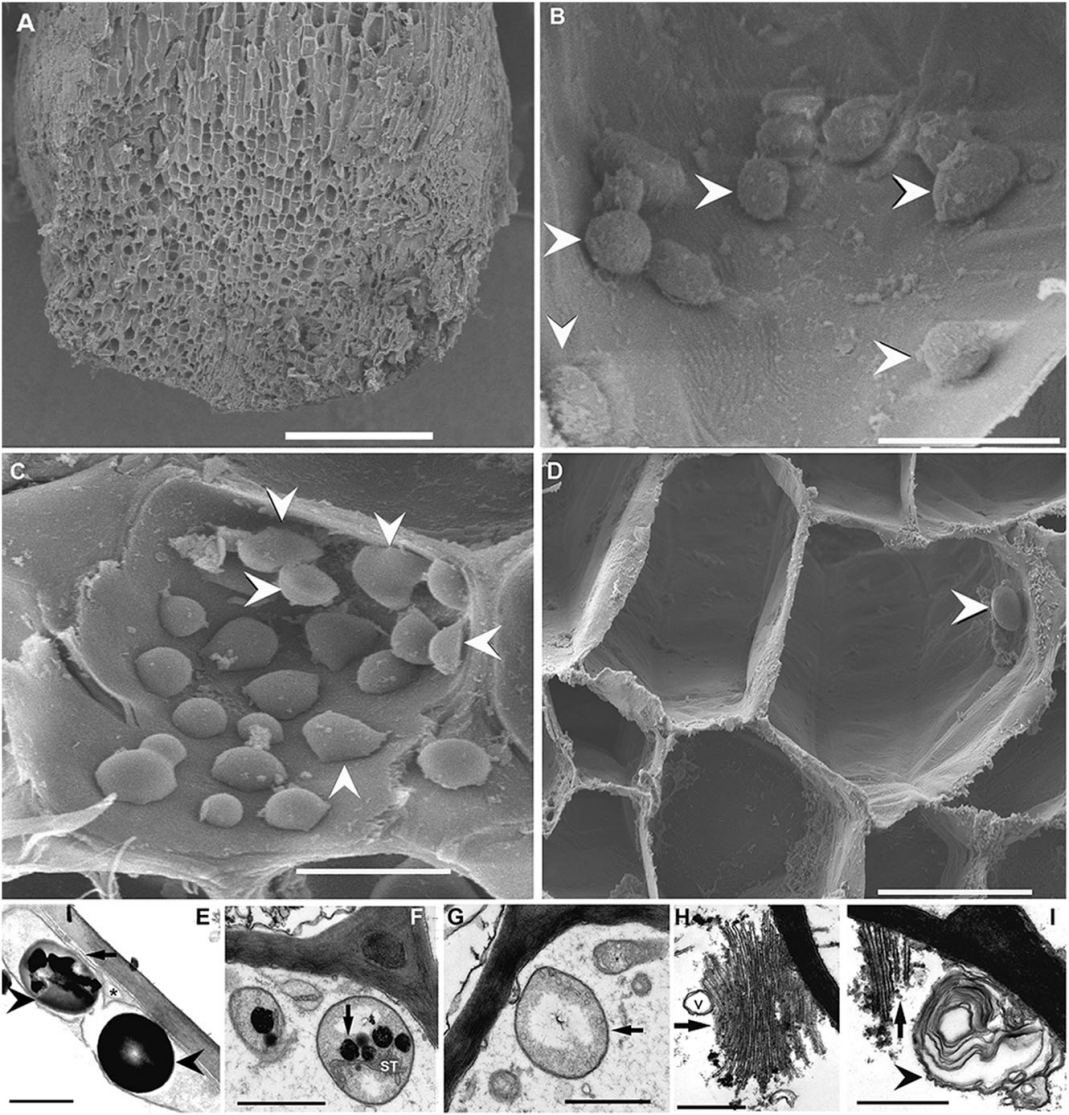
*Posidonia oceanica* root. **A**–**D** Scanning electron microscopy (SEM). **E**–**I** Transmission electron microscopy (TEM). **A**, **B**, **E**, **H** Control (at 120 h after starting the experiment). **C**, **F** Forty-eight hours after sound exposure. **D**, **G**, **I** One-hundred-and-twenty hours after sound exposure. **A** Transverse section of an apical portion of *P. oceanica* root showing the opened root cap containing the columella cells. **B** Opened columella cell showing normally shaped inner starch grains (arrowheads). **C** Some starch grains presented a deformed structure without the typical spherical shape (arrowheads). **D** The number of inner starch grains in columella cells dramatically decreased. Only one remaining starch grain is visible (arrowhead). **E** Amyloplasts containing prominent starch grains (arrowheads). Note the association of endoplasmic reticulum cisternae (arrow) with the amyloplasts and the presence of a vesicle (asterisk). **F** Absence of starch grains and presence of plastoglobuli (arrow) in amyloplasts in close association with stroma thylakoids (ST). **G** Note the absence of starch grains in the amyloplast (arrow). **H** Healthy membranous structure (Golgi complex, arrow) in columella cells (v: vesicle). **I** Myelin-like formation (arrowhead), provoked by sound exposure. The arrow shows the leftover of a membranous structure. Scale bar: **A** = 0.5 mm. **D** = 30 μm. **B**, **C** = 10 µm. **E**–**I** = 1 µm.

In plants, sound can offer an effective channel for short-range signaling, possibly involved in modulating root growth^23^. Columella cells, located in the inner, central region of the root cap, play a fundamental role in gravitropism. These cells show structural polarity, characterized by the position of a nucleus in the proximal part and numerous starch-filled plastids (amyloplasts) and endoplasmic reticulum membranes in the distal part^24^. Amyloplasts are plastids (cytoplasmic organelles surrounded by a double-lipid membrane, with their own DNA and independent replication) that produce and store starch inside the internal membrane compartments^25^ (Fig. 1) and are involved in gravity sensing^24,26^. The sedimentation of these dense amyloplasts, with their inner starch statolith, triggers gravity signal transduction. This signal is transmitted (in a process that involves auxin transport) to the elongation zone, where it promotes differential cell growth, allowing the root to direct itself downward^24^. Gravisensory ion channels and cascades of second messengers, as well as cytoskeletal elements, are involved in the complex process of gravity sensing and graviorientation^24^.

Green plants produce starch for energy storage over long periods. In addition to root cap columella cells^24^, *P. oceanica* presents starch-containing amyloplasts in the rhizome cortical cells^27^. The amyloplast role in orienting the plant in the water column^24^ is analogous to that of sound-sensitive statocysts in marine invertebrates^8,11,12,28^. The amyloplasts would operate like statocysts in *P. oceanica* roots and rhizomes^24^ and probably have evolved to have a role in sound and vibration reception^23^.

Sound perception in plants is an incipient field of research. Although some studies have shown evidence of terrestrial plant’s capacity to acquire and respond to acoustic information^23^, none has shown ultrastructural damage after sound exposure: our results reveal that aquatic plants present acoustic trauma in roots and rhizome cells when exposed to noise. The selection of rhizome cortical cells and root cap collumella cells as metrics to analyze the sound impact in *P.oceanica* was motivated by the presence of amyloplast in both of them. Amyloplasts have evolved as analogs of the invertebrate statocysts^24^, sensory organs responsible for gravity perception, which showed to be sensitive to noise^29^. In addition, we found a degradation in the specific fungal symbionts of *P. oceanica* roots.

## Results

### Scanning and transmission electron microscopy (SEM–TEM)

**Fig. 2:**
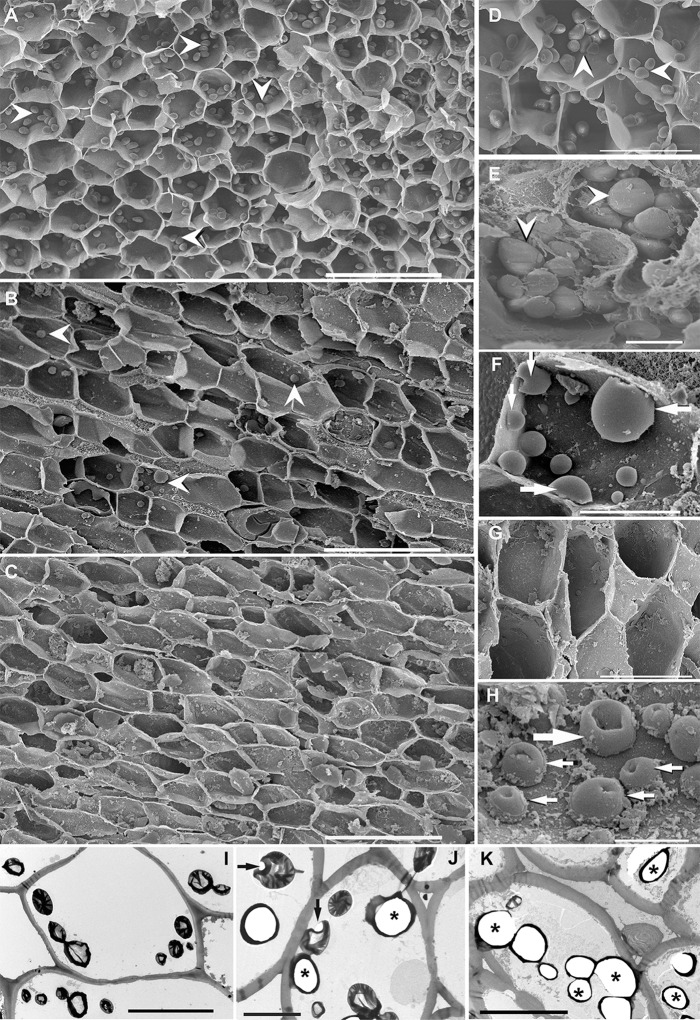
*Posidonia oceanica* rhizome. **A**–**H** Scanning electron microscopy (SEM). **I**–**J** Transmission electron microscopy (TEM). Transverse section of an apical portion of *P. oceanica* rhizome showing the opened cortical cells. **A**, **D**, **E**, **I** Control at 120 h after sound exposure. **B**, **F**, **J** Forty-eight hours after sound exposure. H Ninety-six hours after sound exposure C, G, K One-hundred-and-twenty hours after sound exposure. **A** Cortical cells present a high density of starch grains (arrowheads). **B** The number of starch grains (arrowheads) had considerably decreased. **C** Cortical cells have totally lost the starch grains. **D**, **E** Details of A, showing well-formed (or round) starch grains (arrowheads). **F**, **H** Details from B. **F** Some starch grains presented a deformed structure without the typical spherical shape (arrows). **G** Detail from C, showing the empty columella cells. **H** The remaining abnormal starch grains presenting an empty aspect (large arrow) or holes (small arrows) at the surface. **I** All cortical cells of control rhizomes contained starch-prominent starch grains. **J** Some of the starch grains had partially (arrows) or totally (asterisk) lost the starch. **K** All of the starch grains presented an empty structure (asterisk). Scale bar: **A**–**C** = 100 μm. **D**, **G** = 50 μm. **F**, **I**, **J**, **K** = 20 μm. **E** = 10 μm. **H** = 5 μm.

After exposing *P. oceanica* to low-frequency sounds, we monitored the morphological and ultrastructural effects on root and rhizome amyloplasts. We found that the number of starch granules had progressively decreased after the exposure until their total disappearance (Figs. 1, 2). All control plants (Fig. 1A, B, E, H; 2A, D, E, I) showed a high number of starch-containing amyloplasts, whereas all exposed plants (Fig. 1 D, F, G, I; 2B, C, F–H, J, K) showed a decreasing number of starch grains with time. At 48 h after sound exposure (sweep 50–400 Hz, see “Materials and methods”), some starch grains presented a deformed structure (Figs. 1C, 2F) lacking the typical spherical shape. In some cases (96–120 h after exposure), the remaining starch grains presented several holes at the surface and a deformed organelle structure, probably because of the expulsion of its inner material (Fig. 2H). TEM also showed the presence of plastoglobuli (Fig. 1F) and myelin-like features as a massive accumulation of membranes, potentially created by vacuolar fragmentation or endoplasmic reticulum disorganization (Fig. 1I) induced by the sound exposure.

**Fig. 3:**
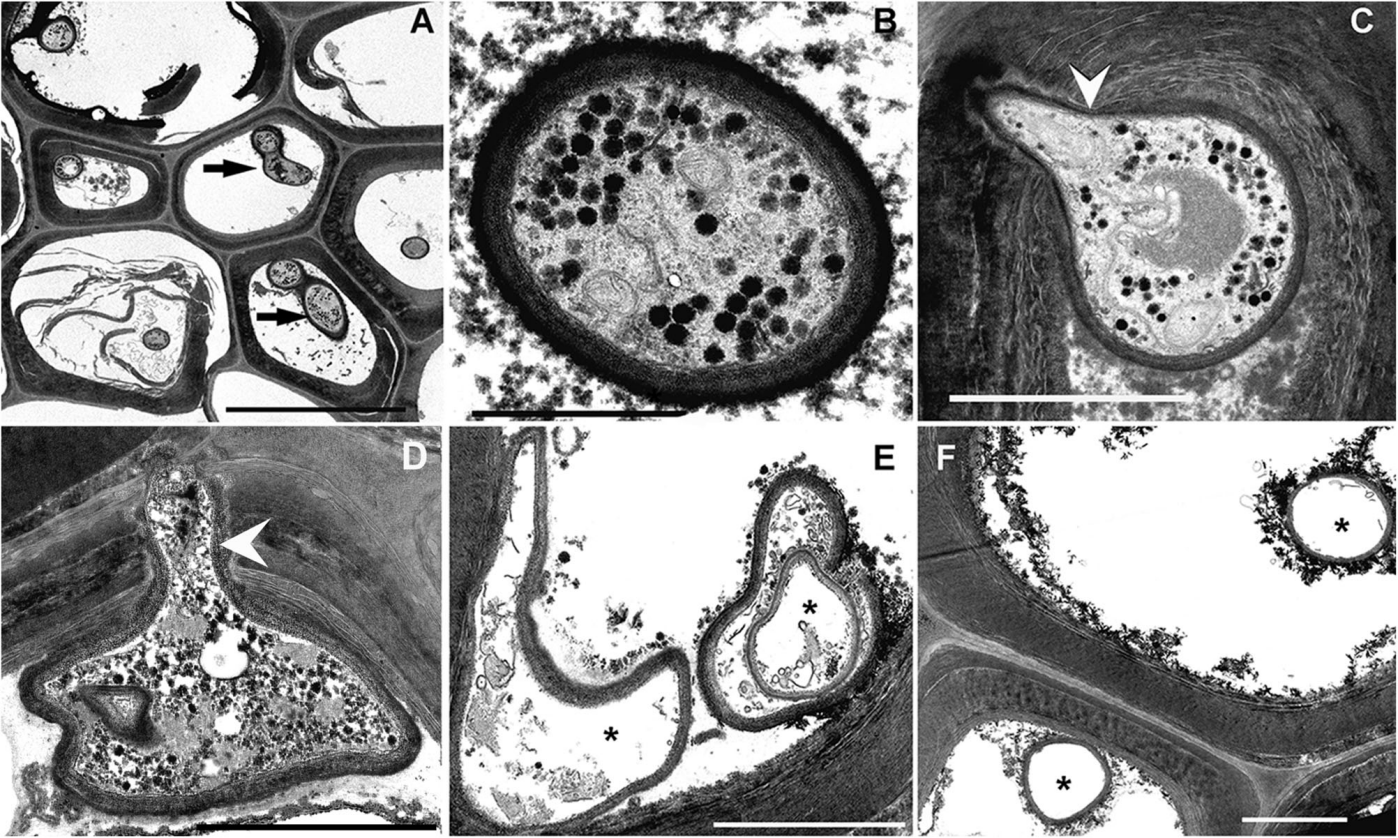
Transverse section through a *P. oceanica* root showing fungal colonization. Transmission electron microscopy (TEM). **A**, **B**, **C** Control at 120 h after starting the experiment. **D** Forty-eight hours after sound exposure. **E** Ninety-six hours after sound exposure. **F** One-hundred-and-twenty hours after sound exposure. **A** Hypodermal cells are colonized by numerous intracellular hyphae (arrows). **B**, **C** The intracellular hyphae present a healthy aspect showing an organelle-rich cytoplasm. **C**, **D** An intracellular hyphae degrading the cell wall while passing from one cell to another (arrowhead). **D** The cytoplasm of the hypha starts to present small vacuoles and fragmented organelles. **E** The intracellular hyphal spaces presenting an almost empty cytoplasm (asterisks). **F** Hyphae presenting a totally empty intracellular space without organelles (asterisks). Scale bar: **A** = 10 μm. **B** = 1 μm. **C**–**F** = 2 μm.

The fungal hyphae colonizing hypodermal cells of exposed *P. oceanica* roots (Fig. 3D–F) presented a degraded aspect compared to control roots (Fig. 3A–C). The cytoplasm of the exposed hyphae showed a progressive alteration of the intracellular organelles with time, reaching a totally empty cytoplasm at 120 h after sound exposure.

### Analysis of starch grain count

**Fig. 4:**
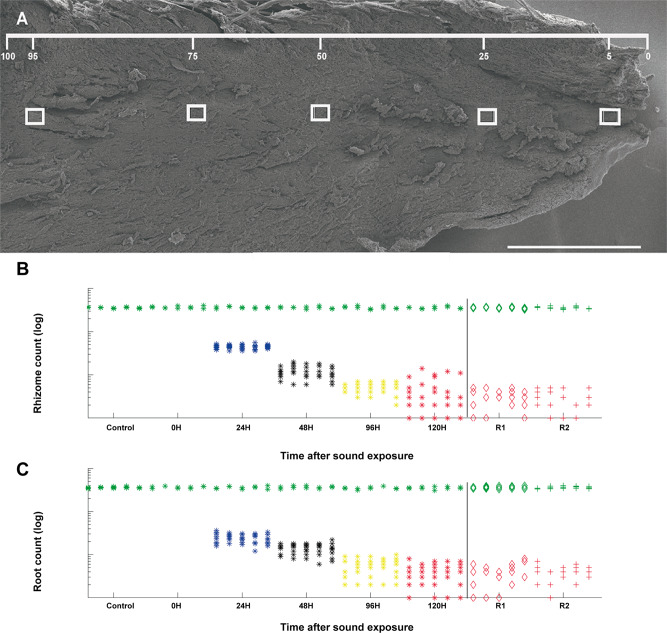
Count locations on the *P. oceanica* rhizome. Starch grain count of the rhizome and root samples. **A** Count locations on the *P. oceanica* rhizome. Starch grain counts were sampled at five predetermined locations: 5%, 25%, 50%, 75%, and 95% of the total sampling zone length. A In all, 0.09-mm^2^ (300 × 300 μm) box was placed at each sampling area, and starch grains were counted within each box. Scale bar = 3 mm. **B**, **C** Results of the starch grain count of the rhizome samples (B) and root samples (C). The left part shows the results of the initial experiments. PRE counts were taken from samples that were fixed immediately after plants arrived in the laboratory. At 0H, the sound exposure started and a control sample was taken before exposure. At each further sampling moment, two control samples were taken together with exposed samples. R1 and R2 are the two replicates (control in green) at 120 h after exposure (in red). The y-axis is shown in logarithmic scale to reduce the order-of-magnitude difference between control and exposed samples. For the exposed samples, the counts are organized per sampling time (hours after exposure) and within each sampling time per sampled area (5–95%). For the control samples, all sampling times were combined together and the samples are separated per sampled area.

We summed the grain count over all five regions (count locations, Fig. 4A) to simplify the comparison between different time delays. Therefore, we looked at the percentage of grains (over total per sample grain count) in each region of the control samples. The rhizome control samples gave the following grain percentages per region (5–95% region): 20%, 20%, 19%, 21%, and 19%. Similarly, the root control samples gave per region grain count percentages (5–95% region): 19%, 20%, 20%, 20%, and 20%. From this, we concluded that the grain counts or densities were similar between regions and weighing factors were not necessary to sum grain counts over regions.

To evaluate if the exposure affected the total grain count in the rhizome samples, we took the sum of the grains over the five regions for each sample. To limit the number of tests, we used the Wilcoxon rank-sum test to compare the median total grain count between control and 24 h, 24 and 48 h, 48 and 96 h, and 96 and 120 h. A visual comparison between the results from controls, 24-h and 48-h counts, made it quite clear that there was a deterioration in the grain count. We chose to use right-tailed tests to test for an increasingly lower grain count rather than simply a change in the count (however, all p-values are provided here to assess the significance). The median grain counts and dispersion can be found in Table 1. In all cases, the alternative hypothesis was that the median grain count of the first set was higher than the median of the second set (right-tailed test). The null hypothesis was rejected in all tests. It is noted that the difference in dispersion of the measurements between control and 24 h is particularly large, preferring an interpretation in terms of stochastic dominance instead of the difference in the median, where control dominates 24 h. After exposure and after each longer time delay, the grain density was significantly lower. For the root comparison, a similar result was found rejecting the null hypothesis in all cases (Table 1). As with the rhizome tests above, when there was a large difference in dispersion, the test result was interpreted in terms of stochastic dominance.

**Table 1:** . Median and median absolute deviation of grain counts with comparison test results.

Exposure Time Comparison					
RHIZOME					
grain count	control	24h	48h	96h	120h
median	1796	223	59	26	12
MAD	14	4	5	1.5	3
rank sum test	control vs 24h	24h vs 48h	48h vs 96h	96h vs 120h	
p-value	3.1e-6	9.1e-5	8.9e-5	2.2e-5	
Z	4.52	3.74	3.75	4.08	
r	0.77	0.84	0.84	0.75	
ROOT					
grain count	control	24h	48h	96h	120h
median	1802	119	70	26	16
MAD	23	7.5	1.0	1.0	1.0
rank sum test	control vs 24h	24h vs 48h	48h vs 96h	96h vs 120h	
p-value	3.1e-6	8.5e-5	8.2e-5	4.8e-6	
Z	4.52	3.76	3.77	4.43	
r	0.77	0.84	0.84	0.81	
**Affected Region Comparison**					
RHIZOME					
region	5%	25%	50%	75%	95%
median	0.20	0.20	0.20	0.18	0.19
MAD	0.034	0.058	0.054	0.042	0.043
ROOT					
region	5%	25%	50%	75%	95%
median	0.21	0.21	0.20	0.23	0.21
MAD	0.062	0.045	0.043	0.058	0.067

Z: test statistic - Wilcoxon rank sum test. r: effects size MAD: median absolute deviation

Finally, we tested if the treated samples were affected differently in different regions, i.e., if one region was more sensitive to the exposed frequencies than the others. The Kruskal–Wallis test was used on the five different regions, first on the rhizome data to test for a difference in the median between all the regions. The null hypothesis could not be rejected with *p* = 0.34 (see Table 1 for the median and MAD values). The distribution of grains per region closely followed the distribution of the control data with about 20% of the grains in each of the five regions after treatment. The results indicated that none of the regions of the rhizome showed a particular lower or higher sensitivity to the exposed frequencies. For the root analysis, we reached the same conclusion, with *p* = 0.68.

The starch-grain density decreased significantly and progressively after exposure (see Fig. 4 and Supplementary Data S1, S2). The results indicated that none of the observed regions of the rhizome and roots showed a particular lower or higher sensitivity to the exposed frequencies.

## Discussion

The almost complete loss or deformation of the starch grains provides evidence that the induced damage was acute and immediate, significantly affecting the plant’s cellular organelles, specifically the amyloplasts. As a consequence, the correct function of the gravitropism process mediated by these organelles could be altered. In addition, the decrease of the amyloplast number could affect the rhizome storage capacity.

In mammals, an acoustic trauma in the organ of Corti—responsible for the transduction of auditory signals in the mammalian ear—alters the surface cytoarchitectural organization and actin arrangement^30^. After sound exposure, invertebrates show a downregulation of cytoskeletal proteins and other proteins related to the microtubular structures of the statocyst sensory epithelia^13^. Organelles in plant cells are positioned and arranged by actin microfilaments^24,26^, while amyloplast (i.e., plastids that produce and store starch) movement is an intricate process involving the vacuolar membrane and actin cytoskeleton. Actin microfilaments may form a sieve-like network that facilitates the movement of the amyloplasts. They could also regulate gravity sensing if they bind to or help lift amyloplasts of the cell floor. The possibility that the actin cytoskeleton is also involved in the process of signal transduction in plants has been proposed^24^. In that scenario, a possible distortion of the anchorage of the starch grains into the vegetal cell, resulting from changes in actin arrangements after sound exposure, could explain the dramatic decrease in their number. In animals, the damage induced by noise and ototoxic drugs (medications that damage the ear, resulting in hearing loss or balance disorders) tends to share common features in injured sensory cells^29^. These features are essentially linked to an apoptotic process and thus can be unspecific to these two modes of injury, but are not attributed to any other external agent. In other words, the origin of the observed lesions can only be attributed to sound or ototoxic drugs. Although there is no evidence that ototoxic drugs can have any effect on plants, their absence in any case in the experimental tank is consistent with the statement that the origin of the observed lesions is noise exposure. In addition, rapid action on the actin cytoskeleton of hair cell stereocilia and cell body is only restricted to acoustic trauma. Noise is known to induce depolymerization of actin filaments and a loss of cross-bridges between filaments in cochlear cells^31–33^. This trauma caused by noise exposure is consistent with our results.

Alternative or additional molecular mechanisms could be involved in sound-induced amyloplast damage. Interestingly, in animal auditory hair cells, loud noise exposure appears to primarily impact double-bound organelles involved in energy- storage-production, e.g., mitochondria^34^. In *P. oceanica*, the organelles responsible for energy storage are amyloplasts, which also present a double membrane. In mammal acoustic trauma, mitochondria trigger cell death pathways in auditory hair cells. This action is likely to be mediated by calcium overload^35^ and an overproduction of mitochondria ROS (reactive oxygen species)^36^. Sound stimulation produces high metabolic demands of the cochlea mechanosensory hair cells. After noise overstimulation or ototoxic drug exposure, hair cell mitochondria produce ROS in excess. This oxidative stress insults the hair cell antioxidant defenses, causing genetic and cellular alterations that produce cellular dysfunctions and lead to a permanent cochlear degeneration^36^.

A possible model of the plant stimulation by sound vibrations would imply a cascade of a signaling process where Ca^2+^ would be a candidate second messenger. In the cells that are exposed to sounds, the production of ROS and proline would increase, thus facilitating a greater activation of Ca^2+^ and K^+^ channels^37^. Calcium and oxidative stress could be involved in the *P. oceanica* root and rhizome statocyst alteration after sound exposure.

Several studies of shoot statocysts of *Arabidopsis* indicate that a functional interaction between cell‐filling vacuoles and adjacent actin microfilaments is also important for controlling amyloplast status and movement^24^. In contrast, vacuoles do not fully occupy the cytoplasm of root columella cells, which instead have only several small vacuoles. Treatments with drugs, such as the actin-depolymerizing latrunculin B, cause vacuolar fragmentation, vacuolar dynamics deficiency, or endoplasmic reticulum disorganization in several types of plant cells, creating myelin-like formations, i.e., a massive accumulation of membranes that disrupt amyloplast dynamics^24^. Here, we observed a similar cell organelle breakdown (Fig. 1I) that could result in (i) a dysfunction of the normal gravitropism from amyloplast dynamics disruption, and (ii) a modification of the rhizome storage function, which is fundamental for seagrass survival.

The presence of plastoglobuli in amyloplasts of the columella cells of exposed roots can also constitute an indication of a sound exposure effect. Plastoglobuli are plastid lipoprotein particles surrounded by a lipid monolayer membrane containing small specialized hydrophobic molecules^38^. These particles have a remarkably dynamic nature. They can rapidly increase or decrease in size during developmental transitions or in response to changes in environmental conditions, such as droughts, high-light stress, or nitrogen-limiting conditions. TEM observation indicates a metabolite exchange between the thylakoid membrane and plastoglobuli along with possible de novo metabolite synthesis and export^38^. This mechanism could explain why we find this organelle in contact with stroma thylakoids in our exposed specimens (Fig. 1F), whereas these were not connected in control specimens after similar amount of time had lapsed. Plastoglobuli are lipid microcompartments involved in plastid metabolism that play a fundamental role in redox regulation, plastid biogenesis, senescence, and environmental adaptation^38^. The presence of these organelles in our exposed specimens could indicate the starting of processes associated with plastid biogenesis or repairing mechanisms at the cellular level in response to the oxidative stress triggered by ROS overproduction after sound exposure.

*P. oceanica* roots are known to be colonized by symbiotic melanized septate hyphae^39^ (similar to the dark septate endophytes present in roots of most terrestrial plants). The biological significance of this fungus–plant symbiosis remains unknown. In terrestrial ecosystems, the vascular plants form dual root-fungus organs called mycorrhizae. In this symbiotic association, mycorrhizal fungi improve the nutrient status of their host plants (mineral nutrition, water absorption, growth, and disease resistance), in exchange for photosynthetic carbon necessary for fungal growth and reproduction^40,41^^.^ The presence of symbiotic melanized septate hyphae in *P. oceanica* roots may suggest an involvement of the specific root symbiosis in the nutrient uptake by this seagrass^42^. Its presence in *P. oceanica* roots in the NW Mediterranean Sea and not in other species of seagrasses, however, indicates a special relationship between this dominant Mediterranean seagrass and its dark septate mycobionts^39^. In addition, to gravireceptor malfunctioning because of a reported loss of starch statocyst, the degradation of the specific fungal symbionts of *P. oceanica* roots after sound exposure could induce further disruption to normal root function. Such damage could affect seagrass by altering its life-supporting nutritional processes.

Ocean soundscapes are integrated by the combination of biological (biophony), geophysical (geophony), and anthropogenic (anthrophony) sounds produced from a variety of sources^43,44^. These three components interact with each other and determine the underwater sound signatures^45^, for each ecosystem showing its daily or seasonal pattern^46^. As an example, the circadian and seasonal soundscape of the shallow waters of a Mediterranean Sea-protected marine area^47^ shows that low frequencies (<1 kHz) are dominated by noise generated by waves (geophony). Higher frequencies (4–96 kHz) are dominated by marine invertebrates (particularly, snapping shrimp), especially at dusk. Fish tend to vocalize at sunset, producing sounds below 2 kHz. Human noise is generated through all at-sea operations and the frequency components concentrate most of their energy below 500 Hz. The choice of the acoustic parameters used in the experiments was based on the necessity to test a broad range of frequencies on a limited number of samples. In addition, the chosen sweep included most of the acoustic components commonly found associated with coastal human activities. This protocol was successfully followed in previous experiments involving marine invertebrates^11,12,29^.

Industrial offshore operations, including renewable energy construction, introduce sources of artificial noise in natural habitats where a diversity of marine species can be found. Sessile organisms do not have the same capacity to escape from insonified areas as mobile creatures do. The results presented here reveal the sensitivity of aquatic plants to sound that may carry a direct consequence on marine biodiversity and may contribute to the increasing fragility of seagrass meadows. Whether the exposure to noise affects plants (including other smaller seagrass species) that are already rooted or juvenile stages that require finding the ground to root themselves, requires further investigation. These findings may contribute to grant new permits that would allow to undertake more complex experiments on a larger number of samples. In particular, these experiments should aim at clarifying the contribution of particle motion components to the sound sensitivity of *P. oceanica* and help determine exposure threshold levels. Certainly, more complex techniques of analysis are now needed to complete our understanding of aquatic plant sensitivity to noise processes. Meanwhile, in *P. oceanica* these results do not only demonstrate that plants are sensitive to noise but that human-generated noise may contribute in the future to the depletion of seagrass meadows due to the increase of human noise introduction in all oceans of the world. The human industries, particularly the marine renewable energy operators and maritime transports, must play a leading role in adopting a responsible environmental approach that requires considering the potential effects of noise at an ecosystem level wherever an operation is planned.

We consider that these results come at a crucial moment when regulators must be provided with data to embrace the full picture of how ocean life is responding to artificial acoustic pressure. These results encourage further investigation on sessile organisms before the first tolerance thresholds are established to reach a good environmental status of the seas.

## Materials and methods

### Plant material

*P. oceanica* is a protected species and as such no invasive experiments are allowed at sea. However, a few individuals can be collected under very strict government control. In this study, samples were collected from *P. oceanica* meadow off the coast of Catalonia, Spain (NW Mediterranean Sea, latitude 41°09’352 N longitude 1°43’651 E), at 175-m depth (shoot density: 221 ± 16/m^2^.Total foliar surface: 264 ± 25 cm^2^/shoot) in August 2018 and in July 2020 (DG051201-215/2018 authorization, Ministry of Agriculture, Livestock, Fisheries and Food—Generalitat de Catalunya). Plant material consisted of 62 (experiments) and 22 (replicates) plants in total, including *n* = 52 and *n* = 22 for SEM, and *n* = 10 for TEM. The plants were transported in seawater to the laboratory facilities and maintained in sand sediment for about four weeks (26 days: T1–T26) in a closed system of recirculating natural seawater (at 22–26 °C, salinity 35 ppt, natural oxygen pressure and main natural lighting average of 500 µmol photons m-2 s^−1^ irradiance on a 14-h photoperiod) consisting of two mechanically filtered (physicochemical self-filtration system with activated carbon and sand, driven by a circulation pump) fiberglass-reinforced plastic tanks with a capacity of 2000 L each and connected to each other^29^. Illuminance and temperature were monitored continuously with an underwater HOBO Pendant® Temperature/Light Data Logger (Onset Corporation, USA). Two controls were taken and analyzed at each arrival of the plants at the laboratory facilities and at the start of each experiment and replicate. Controlled exposure experiments started at T21. The adaptation of the plants to laboratory conditions was assessed at T21 by looking at root and rhizome status from controls (SEM *n* = 2, TEM *n* = 1 at T21) and by comparing them with the initial controls (T0). A single rhizome and a single root sample were collected from each plant, i.e., all samples that were observed came from different plants (Table 2 for details).

**Table 2:** Number of plants taken for the analysis.

		(T0)	0 h (T21)		24 h		48 h		96 h		120 h (T26)		Total	
		SEM	SEM	TEM	SEM	TEM	SEM	TEM	SEM	TEM	SEM	TEM	SEM	TEM
Experiment	Exposed (root–rhizome)	–	–	–	*n*=10	*n*=2	*n*=10	*n*=2	*n*=10	*n*=2	*n*=10	*n*=2	*n*=40	*n*=8
	Control (root–rhizome)	*n*=2	*n*=2	*n*=1	*n*=2	–	*n*=2	–	*n*=2	–	*n*=2	*n*=1	*n*=12	*n*=2
	Total												*N*=52	*N*=10
Replicate 1^a^	Exposed (root–rhizome)	–	–								*n*=5		*n*=5	
	Control (root–rhizome)	*n*=2	*n*=2								*n*=2		*n*=6	
	Total												*N*=11	
Replicate 2^a^	Exposed (root–rhizome)	–	–								*n*=5		*n*=5	
	Control (root–rhizome)	*n*=2	*n*=2								*n*=2		*n*=6	
	Total												*N*=11	

*SEM* scanning electron microscopy, *TEM* transmission electron microscopy.

^a^Replicates: To limit the number of samples, lesions were observed after 120 h, when the effects showed to be the most acute in the first set of experiments.

Given that the sample size was limited by administrative constraints, three controlled exposure experiments were performed (one + two replicates). In order to limit the number of experimental plants, the two replicate studies were performed on samples observed at 120 h, since this corresponded to the time in the experiment when the lesions were at their maximum level.

### Sound exposure protocol

**Fig. 5:**
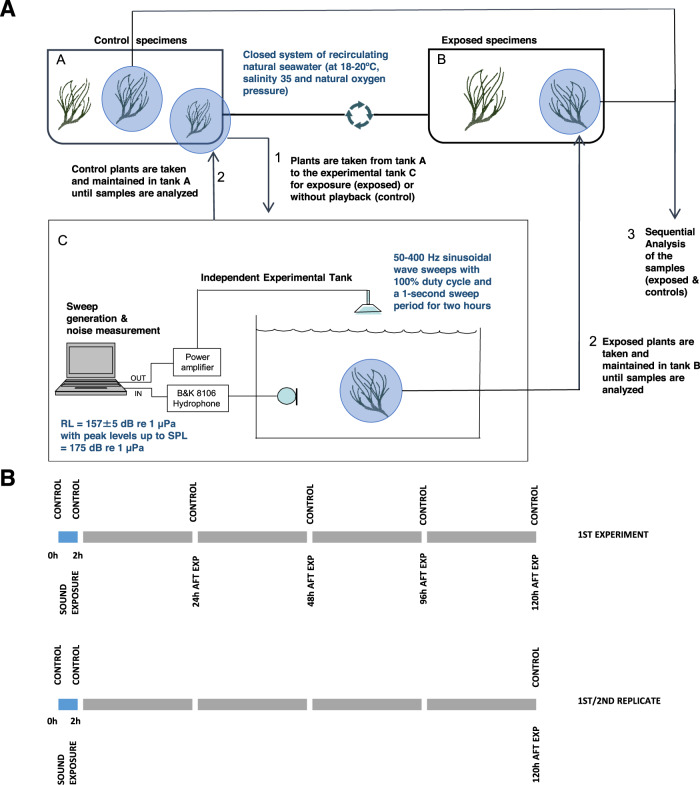
Sound exposure protocol, sampling collection, and analysis. **A** Sound exposure protocol. Plants were maintained in tank A until some were transferred to an independent experimental tank C where they were exposed to sound (1). At the end of the exposure experiments, the plants were transferred to tank B (2) that presented the same environmental conditions as tank A. Control specimens were transferred to tank C for 2 h without any playback and after that, they were taken back to tank A (2). Samples of control and exposed plants were sequentially taken for analysis (3, see Table 2). (Figure modified from^29^). **B** Sampling collection and analysis. Before the sound exposure started, control specimens were taken and analyzed at the arrival of the laboratory facilities and at the beginning of the experiments (T0, T21). Samples of control and exposed plants were sequentially analyzed at 24 h, 48 h, 96 h, and 120 h after sound exposure in the 1^st^ experiment and at 120 h in the replicates (see Table 2).

Sequential controlled exposure experiments were conducted on *P. oceanica* plants (Fig. 5A). The objective of the experiments was to obtain a binary result: does sound negatively affect the plant physiological integrity? The limited sample size allowed only for a small number of experiments. This prevented studying the effects of playback of various sources of anthropogenic noise at sea. As there was no prior information on the sensitivity of aquatic plants to sound, we chose to expose them to a sweep that would cover a large range of frequencies. Although this sweep is not specifically encountered at sea, its frequency components are commonly found to be associated with offshore operations. Following previous publications^29,48,49^_,_ these experiments were not designed to find threshold levels of particular frequencies that would trigger lesions, but to investigate the potential sensitivity of *P. oceanica* to sounds. The need of generating high-amplitude, low-frequency sounds required the use of an in-air loudspeaker, which demonstrated in numerous previous studies to be well suited for underwater-controlled exposure experiments. The sound exposure protocol consisted of sinusoidal wave sweeps of 50–400 Hz with a 100% duty cycle and a 1-second period for 2 h. The level received was measured by a calibrated B&K 8106 hydrophone (sound-pressure levels of 157 ± 5 dB re 1 μPa^2^ with peak levels up to 175 dB re 1 μPa^2^). A study conducted in the same tank conditions with laser Doppler and accelerometer measurements^50^ showed that the distribution of acoustic pressure and particle motion components was heterogeneous. The experimental design was not aimed at mapping this distribution, understanding that at any location in the tank, the plants would be exposed to a contribution of both components of the sound, characteristics of which were not under the scope of this study.

### Sample collection

Apical portions of 10 mm of roots and 15 mm of rhizomes of *P. oceanica* were obtained from the plants (exposed and controls, see Fig. 5B and Table 2) and fixed for ultrastructural analysis (SEM and TEM). For SEM, the PRE data set consisted of two samples taken at the moment of plant arrival in August 2018 and four samples taken on arrival in July 2020. The control samples at 0 h were taken just before each of the three exposure experiments. The other samples were taken at intervals after exposure (24 h, 48 h, 96 h, and 120 h): each time two unexposed control samples and ten exposed samples. For the two replicate studies, only five exposed samples were taken at 120 h.

For TEM, one control sample was taken at 0 h and one at 120 h. The other samples were taken at the same regular intervals as with SEM, with two samples each time. The TEM samples were not used for any statistical tests and no replicate samples were collected.

### SEM

Seventy-four portions of both root and rhizome were used in this study (see Table 2). Fixation was performed in glutaraldehyde 2.5% for 24–48 h at 4 °C. Samples were dehydrated in graded alcohol solutions and critical-point dried with liquid carbon dioxide in a BAL-TEC CPD030 unit (Leica Microsystems, Austria). The dried samples were cut longitudinally to expose the inner root and rhizome structures and then mounted on specimen stubs with double-sided tape and colloidal silver. The mounted tissues were gold-coated with a Q150R S sputter-coated unit (Quorum Technologies, Ltd) and viewed with a variable-pressure Hitachi S3500N scanning electron microscope (Hitachi High Technologies Co., Ltd, Japan) at an accelerating voltage of 5 kV in the Institute of Marine Sciences of the Spanish Research Council facilities.

### TEM

Ten portions of both roots and rhizome were used. Fixation was performed in glutaraldehyde 2.5%–paraformaldehyde 2% for 24 h at 4 °C. Subsequently, the samples were osmicated in 1% osmium tetroxide, dehydrated in acetone, and embedded in Spurr. To orient the specimens properly, semithin sections (1 mm) were cut transversally or tangentially with a glass knife, stained with methylene blue, covered with Durcupan, and observed on an Olympus CX41. Ultrathin (around 100 nm) sections of the samples were then obtained by using a diamond knife (Diatome) with an Ultracut Ultramicrotome from Reichert-Jung. Sections were double-stained with uranyl acetate and lead citrate and viewed with a Jeol JEM 1010 at 80 kV. Images were obtained with a Bioscan camera model 792 (Gatan) at the University of Barcelona technical services.

### Statistics and reproducibility

To quantify the effect of sound on the *P. oceanica*, we considered the whole portion of the rhizome and the region comprising the columella cells in the root cap. The length of the area was determined for each sample, and 0.09-mm^2^ (300 × 300 μm, rhizome) and 0.04-mm^2^ (200 × 200 μm, root) sampling squares were placed along the center length of the area at 5%, 25%, 50%, 75%, and 95% of the long axis of the sample (Fig. 4A).

We looked separately at the grain density in the rhizome and the root. When counting. grains in a specific zone (e.g., 5%, 25%), we made no distinction between normal and. deformed grains and always used the total count (Fig. 4).

In addition to comparing the findings with control samples at each stage of the exposure process, our analysis concentrated on looking at and quantifying how the damage evolved with time after exposure, following a protocol validated in previous studies^11,12,28,29,49^.

All statistical tests were performed in MathWorks MATLAB 2019a using the Wilcoxon rank-sum and the Kruskal–Wallis tests (its extension to multiple groups), comparing the median of grain counts either between the different regions (e.g., 5%, 25%) or between different time delays (e.g., 24 h, 48 h). The significance level for the tests was set to 1%. The dispersion of each group was estimated with the MAD. The effect size r for the Wilcoxon rank-sum test was evaluated by $r=\frac{\mathrm{Z}}{\sqrt{{\mathrm{N}}_{\mathrm{s}}}}$ with Z the test statistic and N_s_ the total number of samples in the two groups being compared. An effect was considered large for r > 0.5, moderate for r > 0.3, and small for r > 0.1. The PRE samples were added to the control data; the two additional replicates for the control data and 120-h time delay were added to the first set of experiment results (*N* = 24 for control, *N* = 20 for 120 h, *N* = 10 for all others).

In order to test if one region was more sensitive to the exposed frequencies than the others, all treated regions from all time delays were grouped together, dividing each region count of a sample by the total grain count of that sample. The normalization was done at this step because we had already determined the relevance of time after exposure and knew that without normalizing, the 24-h data set would dominate the region counts. The Kruskal–Wallis test was used on the five different regions first on the rhizome data to test for a difference in the median between all the regions.

### Reporting summary

Further information on research design is available in the Nature Research Reporting Summary linked to this article.

## Supplementary information

Description of Additional Supplementary Files

Supplementary Data 1

Supplementary Data 2

Reporting Summary

## Data Availability

The authors declare that the data supporting the findings of this study are available within the paper and its Supplementary Data files.
