# Supplementary material for: Seagrass Posidonia is impaired by human-generated noise
Source: Commun Biol. 2021 Jun 15;4:743. doi: 10.1038/s42003-021-02165-3 (PMC8206088; doi:10.1038/s42003-021-02165-3)
Supplement: Supplementary file 1 — Description of Additional Supplementary Files [file 42003_2021_2165_MOESM1_ESM.pdf]

Description of Additional Supplementary Files

File name: Supplementary data 1

Description: ROOT (Number of starch grains). Starch grains counts sampled at different times after sound exposure and at five predetermined locations: 5%, 25%, 50%, 75%, and 95% of the total sampling zone length**.**

File name: Supplementary data 2

Description: RHIZOME (Number starch grains). Starch grains counts sampled at different times after sound exposure and at five predetermined locations: 5%, 25%, 50%, 75%, and 95% of the total sampling zone length.
